# Supplementary material for: Allele-specific differential regulation of monoallelically expressed autosomal genes in the cardiac lineage
Source: Nat Commun. 2022 Oct 10;13:5984. doi: 10.1038/s41467-022-33722-x (PMC9550772; doi:10.1038/s41467-022-33722-x)
Supplement: Supplementary file 1 — Supplementary Information [file 41467_2022_33722_MOESM1_ESM.pdf]

Supplementary Information for:

Allele-specific differential regulation of monoallelically expressed autosomal genes in the cardiac lineage

Gayan I. Balasooriya[1] and David L. Spector[1*]

[1]Cold Spring Harbor Laboratory, One Bungtown Road, Cold Spring Harbor, New York 11724. USA.

[*]Corresponding author: spector@cshl.edu

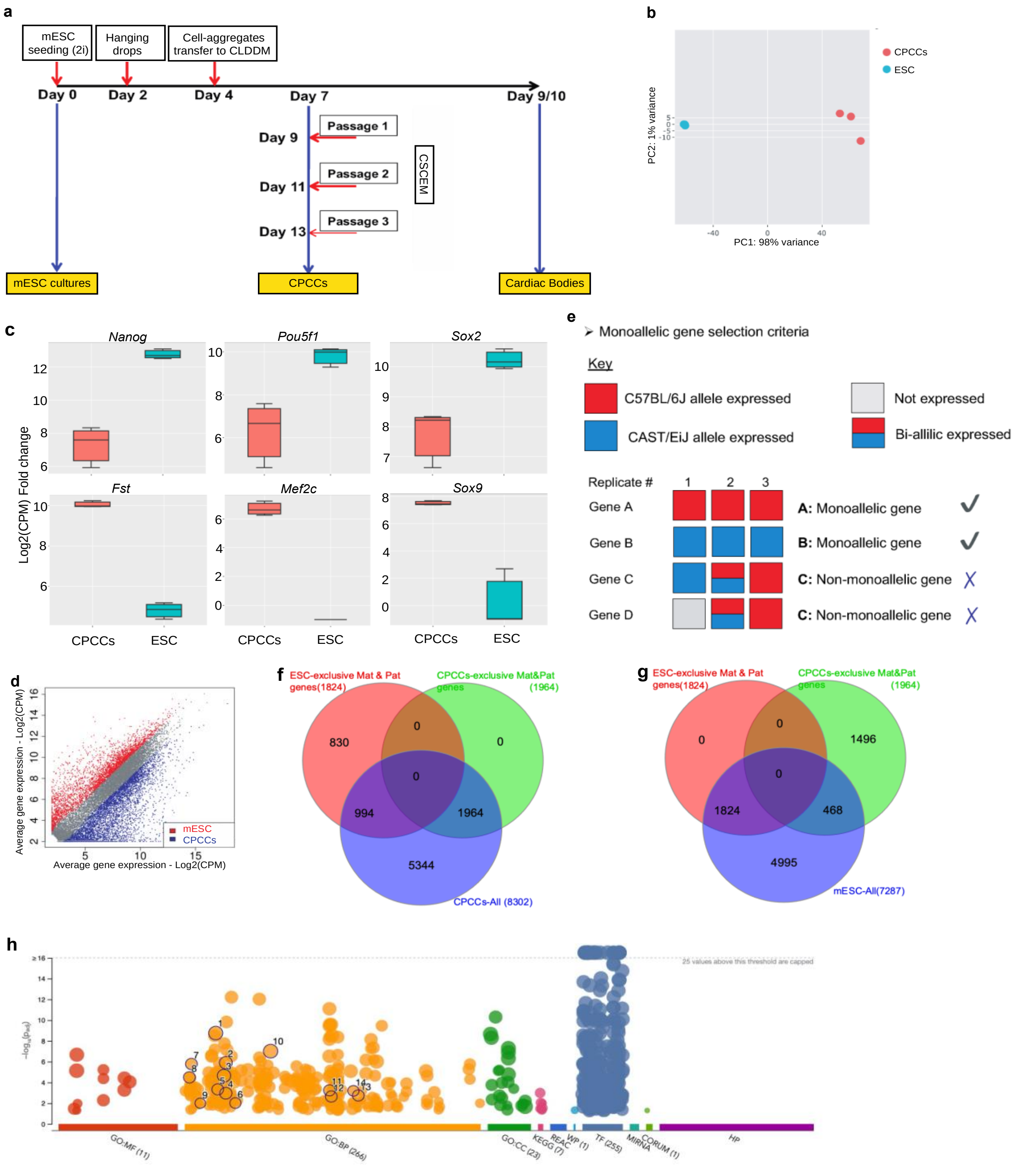

ID	Source	Term ID	Term Name	p_{adj} (query_1)
1	GO:BP	GO:0007275	multicellular organism development	1.754×10^{-9}
2	GO:BP	GO:0009888	tissue development	1.258×10^{-6}
3	GO:BP	GO:0009653	anatomical structure morphogenesis	2.243×10^{-5}
4	GO:BP	GO:0009887	animal organ morphogenesis	1.097×10^{-3}
5	GO:BP	GO:0007507	heart development	4.683×10^{-4}
6	GO:BP	GO:0014706	striated muscle tissue development	9.588×10^{-3}
7	GO:BP	GO:0001944	vasculature development	1.672×10^{-6}
8	GO:BP	GO:0001568	blood vessel development	3.208×10^{-5}
9	GO:BP	GO:0003007	heart morphogenesis	9.984×10^{-3}
10	GO:BP	GO:0032502	developmental process	9.758×10^{-8}
11	GO:BP	GO:0048514	blood vessel morphogenesis	5.740×10^{-4}
12	GO:BP	GO:0048729	tissue morphogenesis	2.266×10^{-3}
13	GO:BP	GO:0061061	muscle structure development	1.778×10^{-3}
14	GO:BP	GO:0060537	muscle tissue development	6.429×10^{-4}

i

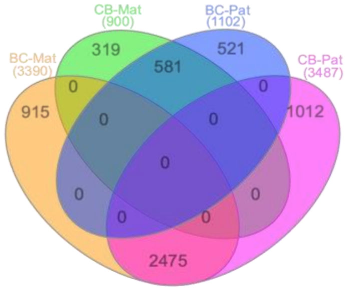

GEO: GSM2405897 (Male)

j

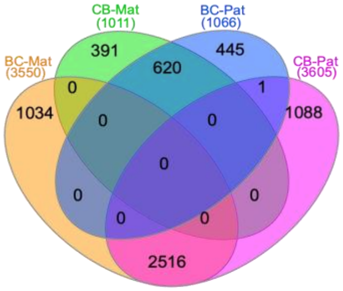

GEO: GSM2405897 (Female)

k

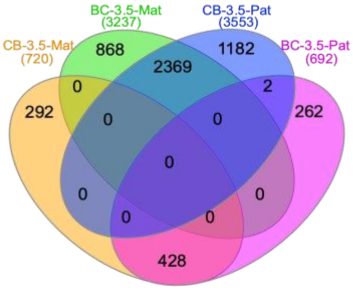

GEO: GSE152103 (Female)

l

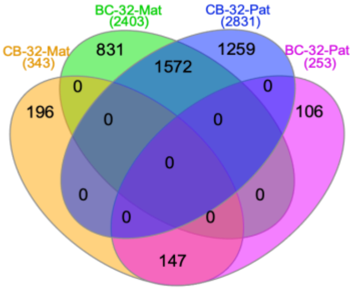

GEO: GSE80810 (Male)

m

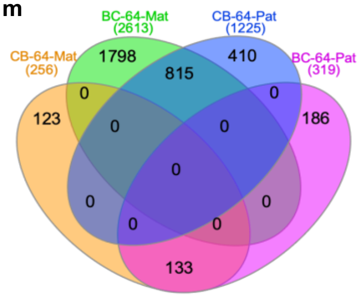

GEO: GSE80810 (Male)

Supplementary Fig. 1| Allelic expression of autosomal monoallelic genes. a, Schematic of in vitro cell differentiation protocol of cardiac lineage cell types. Cardiac intermediate cultures at day 7 are highly heterogenous cell cultures. CSC and CPC enriched cultures were obtained passaging these intermediate cultures three times in enrichment medium before harvesting for RNA-seq experiments. At 9-10 days post ascorbic acid treatment, detached embryonic bodies start rhythmically beating and become cardiac bodies. Picked floating-beating cardiac bodies were used in scRNA-seq experiments. **b**, Unsupervised Principal Component Analysis (PCA) of mESCs and CPCCs bulk-RNA-seq data. **c**, Expression levels of known mESC and CSC-CPC marker genes. Differential gene expression analysis was performed using RNA-seq data count tables from three mESCs and CPCCs biological replicates. Box plots definitions; Nanog: ESC-Max:13.8, Min: 12.98, Median: 12.68, q3: 12.98, q1: 12.55/ CPCCs-Max:8.33, Min: 5.94, Median: 7.6, q3: 8.15, q1: 6.35. Pou5f1: ESC-Max: 10.14, Min: 9.29, Median: 9.99, q3: 10.10, q1: 9.46/ CPCCs-Max:7.59, Min: 4.60, Median: 6.66, q3:7.36, q1: 5.12. Sox2: ESC-Max: 10.6, Min: 9.94, Median:

10.16, q3: 10.49, q1: 9.99/ CPCCs-Max: 8.33, Min: 6.63, Median: 8.21, q3: 8.3, q1: 7.02. Fst: ESC-Max: 5.18, Min: 4.37, Median: 4.86, q3:5.10, q1: 4.49/ CPCCs-Max: 10.25, Min: 9.95, Median: 9.98, q3:10.18, q1:9.96. Mef2c: ESC-Max, Min, Median, q3, q1/ CPCCs-Max: 7.22, Min: 6.24, Median: 6.61, q3: 7.07, q1: 6.33. Sox9: ESC-Max: 2.69, Median: -1.00, q3: 1.17/ CPCCs-Max: 7.67, Min: 7.38, Median: 7.43, q3: 7.61, q1: 7.39. **d**, Differential gene expression in mESCs and CPCCs. Differentially expressed mESC and CPCCs genes are uniquely enriched in each cell type. -Log2(2) CPM was uses as the cut off. **e**, Schematic of the deterministic monoallelic gene selection criteria. Genes are categorized as: not-expressed, maternal-allele only expressed, paternal-allele only expressed and bi-allelically expressed. To be categorized as a monoallelic gene (1), the gene must be expressed in all biological triplicates, and (2) the gene must be exclusively expressed from only one parental background. **f**,**g**, Relation of monoallelic gene establishment between mESCs and CPCCs. Maternal and Paternal DeMA genes in mESCs and CPCCs were assessed among the total CPCCs genes (**f**) and the total mESCs (**g**). Venn diagrams show 994 monoallelically expressed DeMA genes in mESCs changed to biallelic expression in CPCCs and 468 biallelically expressed genes in mESCs changed to monoalleleic expression. **h**, Functional analysis of newly established DeMA genes in CPCCs. GO: BP terms clearly indicate the cardiac lineage specific functional relevance of several new monoallelic genes. The p-value is computed from the Fisher exact test assuming binomial distribution and independence for probability of any gene belonging to any set. **i-m**, Venn diagrams illustrating the effect of genetic background of the alleles in DeMA gene biased maternal and paternal expression. All the analyses show the switch of the DeMA genes depending on the genetic background of the allele. Maternal is abbreviated to "Mat" and paternal is abbreviated to "Pat". CB: cells from CAST/EiJ x C57BL/6J crosses and BC: cells from C57BL/6J x CAST/EiJ crosses. Either in CB or BC, "C" is for CAST/EiJ and "B" is for

C57BL/6J and the first letter indicates the maternal background. GSM2405897: male and female RNA-seq data was generated from mESC bulk cell samples. GSE152103: male and female RNA-seq data was generated from total cells in 3.5 dpc blastocycts. GSE80810: male and female RNA-seq data was generated from single cells of 32-cell and 64-cell blastocyct. Source Data 46-57.

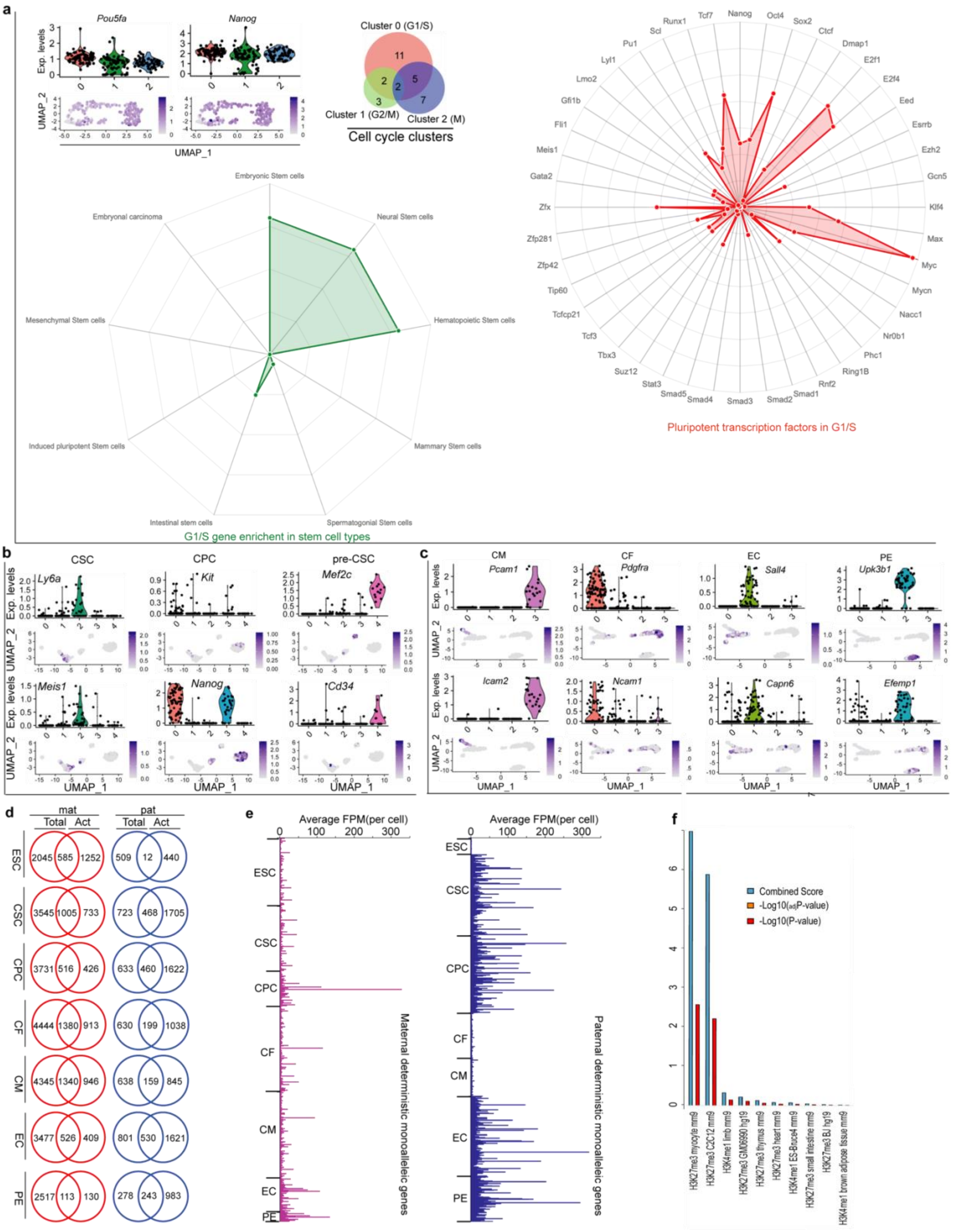
a
Pou5fa
Nanog
Exp. levels
UMAP_2
UMAP_1
Cluster 0 (G1/S)
Cluster 1 (G2/M)
Cluster 2 (M)
Cell cycle clusters
Embryonic Stem cells
Neural Stem cells
Hematopoietic Stem cells
Mammary Stem cells
Spermatogonial Stem cells
Intestinal stem cells
Induced pluripotent Stem cells
Mesenchymal Stem cells
Embryonal carcinoma
G1/S gene enrichent in stem cell types
Pluripotent transcription factors in G1/S
b
CSC
CPC
pre-CSC
Ly6a
Kit
Mef2c
Meis1
Nanog
Cd34
c
CM
CF
EC
PE
Pcam1
Pdgfra
Sall4
Upk3b1
Icam2
Ncam1
Capn6
Efemp1
d
mat
pat
Total
Act
ESC
CSC
CPC
CF
CM
EC
PE
e
Average FPM(per cell)
Maternal deterministic monoalleleic genes
Paternal deterministic monoalleleic genes
f
Combined Score
-Log10(adj P-value)
-Log10(P-value)
H3K27me3 myocyte mm9
H3K27me3 C2C12 mm9
H3K4me1 limb mm9
H3K27me3 GM06990 hg19
H3K27me3 thymus mm9
H3K27me3 heart mm9
H3K4me1 ES-Bruce4 mm9
H3K27me3 small intestine mm9
H3K27me3 BJ hg19
H3K4me1 brown adipose tissue mm9

Supplementary Fig. 2| Allele expression in cardiac cell types. a, mESC cell clustering and cell cycle gene enrichment analysis. Gene expression levels and UMAP plots show high enrichment of *Pou5fa* and *Nanog* in three mESC clusters. Cell cycle transcriptomic analysis project the three cell clusters from three cell cycle stages: Cluster 0: G1/S, Cluster 1: G2/M, Cluster 2: M. Radar-plot showing the gene enrichment in different stem cell types (bottom-left) and key pluripotent transcription factor enrichment in G1/S cluster (bottom-right). **b**, Cell type specific gene enrichment and gene clustering (UMAP-cluster graphs) for CSC, CPC and pre-CSC cells from CPCCs cultures. **c**, Cell type specific gene enrichment and gene clustering (UMAP) for CF, CM, EC and PE in cells from CBs. **d**, Multiple Venn diagram showing the number of genes in two transcript categories, mature (Total) transcripts and nascent (Act) transcripts, for seven cell types studied. Except in mESCs and CMs, the actively expressed number of pat-mono genes is greater than the number of mat-mono actively transcribing genes. Mat-mono genes are lacking their nascent transcripts more often than pat-mono genes. **e**, Average (Fragment Per Million-FPM) maternal and paternal active gene expression for mESCs, CSCs, CPCs, CFs, CMs, ECs and PEs. Data point at the X- axis represent genes. In mESC, CFs and CMs, the transcription rates are higher in mat-mono genes and in CSCs, CPCs, ECs and PEs, the transcription rate is higher in pat-mono genes. **f**, Histone signatures of pat-mono genes. Histone marks enrichment plotted for Combined score, $-\text{Log}_{10}(_{\text{adj}}\text{P-value})$, $-\text{Log}_{10}(\text{P-value})$. The p-value is computed from the Fisher exact test assuming binomial distribution and independence for probability of any gene belonging to any set. Combined score was computed by using the log of the P-value from the Fisher exact test and multiplying that by the z-score of the deviation from the expected rank. Benjamini-Hochberg method was used to correct the multiple hypothesis testing to compute the adjusted p-value.

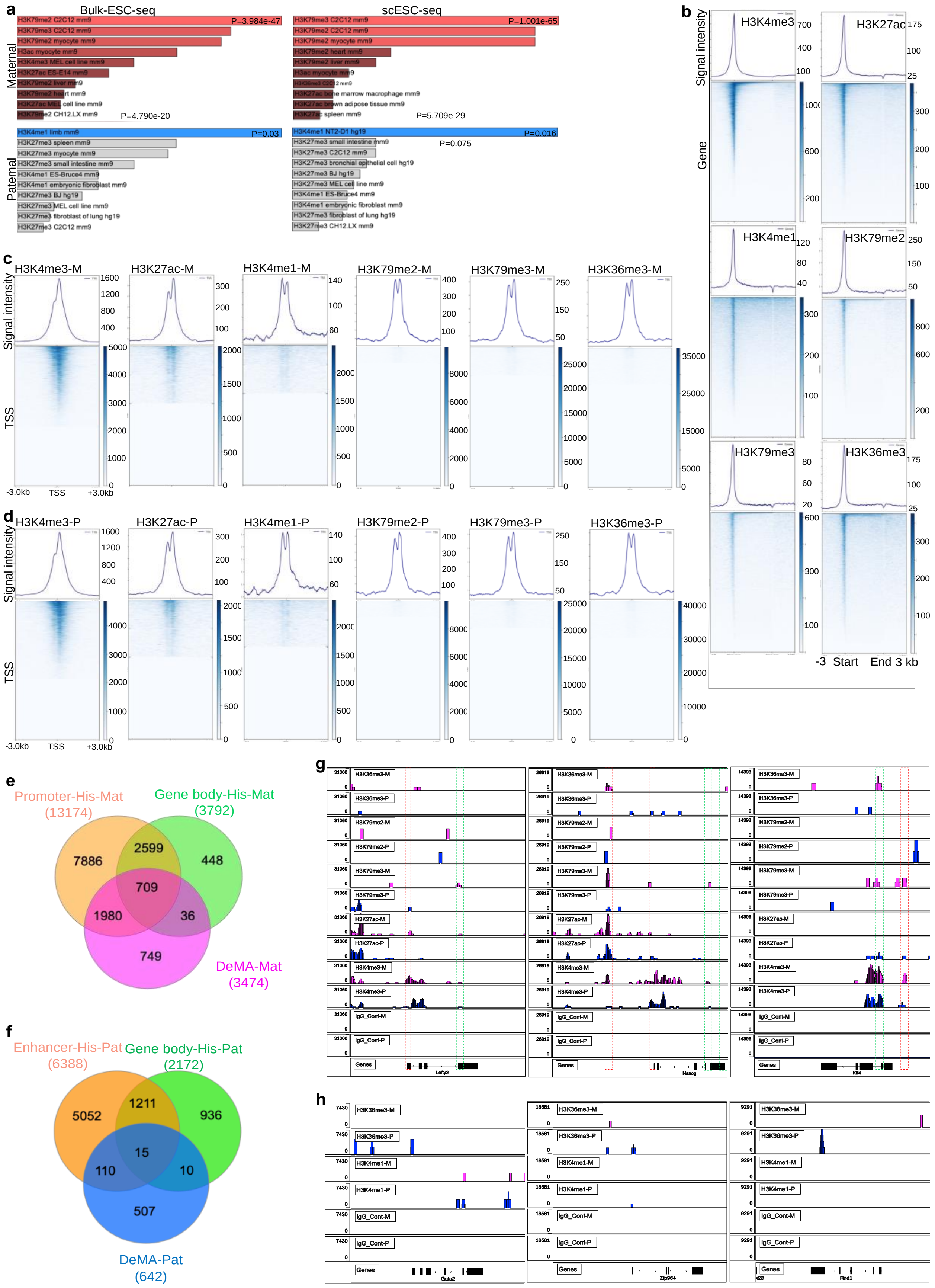

a
Bulk-ESC-seq
scESC-seq
Maternal
Paternal
P=3.984e-47
P=4.790e-20
P=1.001e-65
P=5.709e-29
P=0.03
P=0.016
P=0.075
H3K79me2 C2C12 mm9
H3K79me3 C2C12 mm9
H3K79me2 myocyte mm9
H3K4me1 limb mm9
H3K27me3 spleen mm9
H3K27me3 myocyte mm9
H3K27me3 small intestine mm9
H3K4me1 ES-Bruce4 mm9
H3K4me1 embryonic fibroblast mm9
H3K27me3 BJ hg19
H3K27me3 MEL cell line mm9
H3K27me3 fibroblast of lung hg19
H3K27me3 C2C12 mm9
H3K4me1 NT2-D1 hg19
b
Signal intensity
Gene
H3K4me3
H3K27ac
H3K4me1
H3K79me2
H3K79me3
H3K36me3
-3 Start End 3 kb
c
H3K4me3-M
H3K27ac-M
H3K4me1-M
H3K79me2-M
H3K79me3-M
H3K36me3-M
Signal intensity
TSS
-3.0kb
TSS
+3.0kb
d
H3K4me3-P
H3K27ac-P
H3K4me1-P
H3K79me2-P
H3K79me3-P
H3K36me3-P
Signal intensity
TSS
-3.0kb
TSS
+3.0kb
e
Promoter-His-Mat
(13174)
Gene body-His-Mat
(3792)
DeMA-Mat
(3474)
7886
2599
448
709
1980
36
749
f
Enhancer-His-Pat
(6388)
Gene body-His-Pat
(2172)
DeMA-Pat
(642)
5052
1211
936
15
110
10
507
g
H3K36me3-M
H3K36me3-P
H3K79me2-M
H3K79me2-P
H3K79me3-M
H3K79me3-P
H3K27ac-M
H3K27ac-P
H3K4me3-M
H3K4me3-P
IgG_Cont-M
IgG_Cont-P
Genes
Lefty2
Nanog
Klf4
h
H3K36me3-M
H3K36me3-P
H3K4me1-M
H3K4me1-P
IgG_Cont-M
IgG_Cont-P
Genes
Gata2
Zfp964
Rnd1

Supplementary Fig. 3| Allele-specific differential epigenetic regulation of monoallelic genes. **a**, In-silico analysis of maternal and paternal DeMA genes in F1 mESCs bulk and scRNA-seq data using ENCODE histone data. For maternal genes the curated histone signatures are highly probabilistic, while for the paternal gene the probability of the histone marks curated are comparatively low p=0.03 and p=0.09. The p-value is computed from the Fisher exact test assuming binomial distribution and independence for probability of any gene belonging to any set. **b**, Histone signatures' distribution throughout a hypothetical gene and intergenic regions. **c & d**, Allele specific histone association for the histone marks used in CUT&Tag assay (i-maternal and j-paternal). H3K4me3, a promoter signature indicating the presence of two peaks, a predominant one at the -TSS and a weak peak at the +TSS, most probably an indication of proximal promoters. H3K27ac and H3K4me1, enhancer signatures also indicate two peaks beside the TSS, which may also be associated with proximal enhancers at the region 5' of the TSS. Lower number of gene's 5' and 3' of TSS regions are marked by the H3K79me2, H3K79me3 and H3K36me3 exhibiting active gene promoter association, but the majority of the enrichment is widespread across the gene bodies. **e**,**f** Allele specific histone signatures in F1 mESCs from CUT&Tag assay compared with maternal and paternal DeMA genes. **e**, Histone signatures in promoters (H3Kme3, H3K27ac and H3K79me2) and gene bodies (H3K79me2, H3K79me3 and H3K36me3) of maternal allele comparison with maternal DeMA genes (DeMA-Mat) in RNA-seq data from bulk ESCs. From the total maternal DeMA genes, 78.4% of maternal DeMA genes are exclusively enriched in maternal allele histone signatures. **f**, In line with *in silico* predicted in ENCODE data (at the low probability p=0.03, compared to maternal genes), the paternal DeMA genes (DeMA-Pat) from bulk RNA-seq data from mESCs do not show higher correlation (21%) with enhancer (H3K4me1) or gene body (H3K36me3-form Fig. 2h) association in CUT&Tag data. **g**, Illustration of five allele-specific

histone associations in maternal DeMA genes in mESCs. *Nanog*, *Klf4* and *Lefty2*, known pluripotent associated genes. Red dotted lines indicate the enhancer/promoter regions and green dotted lines indicate the gene body region and histone association. For example, H3K4me3, H3K79me3 and H3K36me3 histone association in ~ +5 kb region of Nanog TSS in maternal allele is shown (*Nanog* is maternally expressed in our study model). **h**, In paternally expressed *Gata2*, *Zfp964* and *Rind1* genes, only the paternal alleles indicate the enhancer and gene body histone signatures. Source data are provided as a Source Data files.

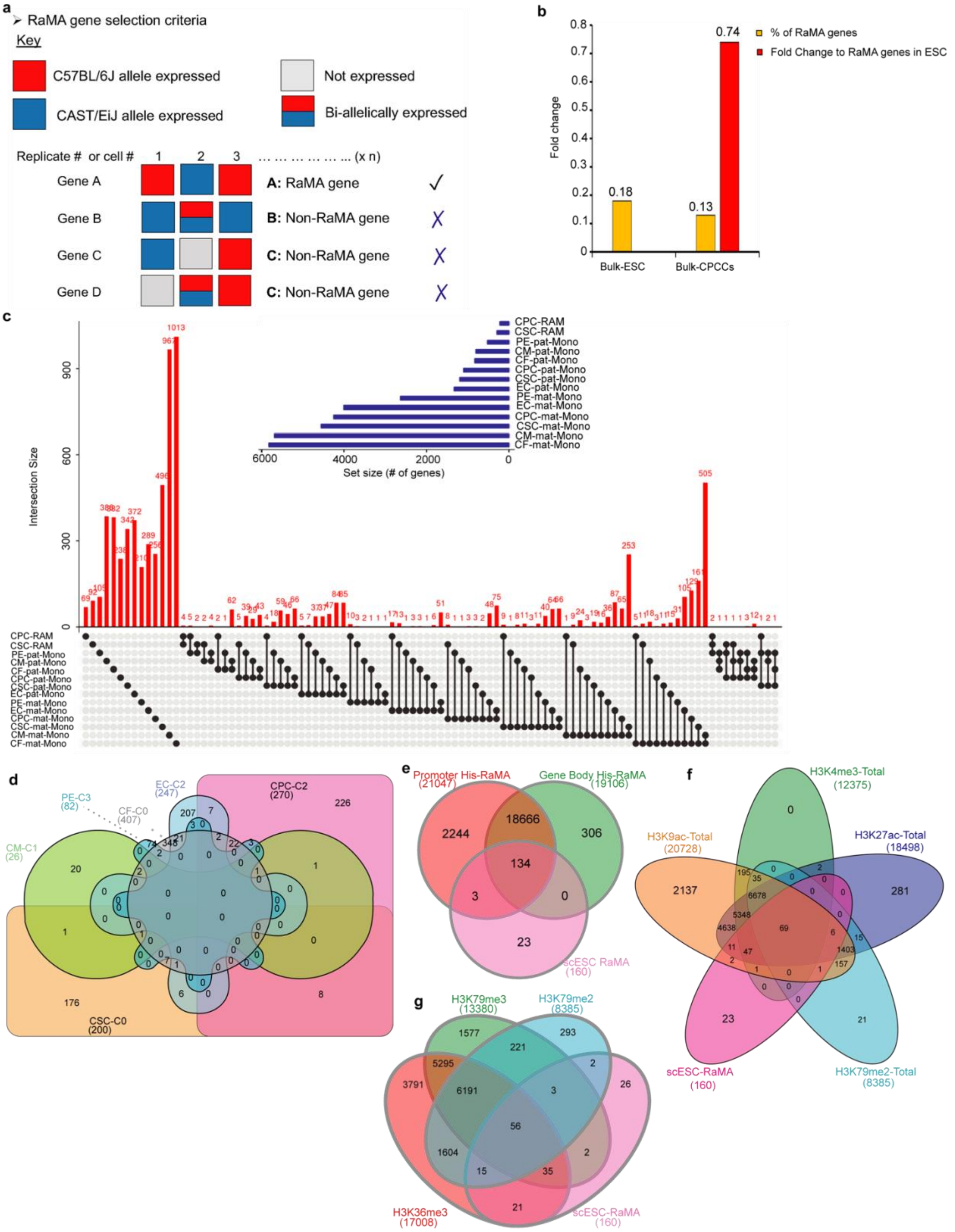
a
RaMA gene selection criteria
Key
C57BL/6J allele expressed
Not expressed
CAST/EiJ allele expressed
Bi-allelically expressed
Replicate # or cell # 1 2 3 … … … … … … (x n)
Gene A
A: RaMA gene
Gene B
B: Non-RaMA gene
Gene C
C: Non-RaMA gene
Gene D
C: Non-RaMA gene
b
% of RaMA genes
Fold Change to RaMA genes in ESC
Fold change
0.18
0.13
0.74
Bulk-ESC
Bulk-CPCCs
c
Intersection Size
Set size (# of genes)
CPC-RAM
CSC-RAM
PE-pat-Mono
CM-pat-Mono
CF-pat-Mono
CPC-pat-Mono
CSC-pat-Mono
EC-pat-Mono
PE-mat-Mono
EC-mat-Mono
CPC-mat-Mono
CSC-mat-Mono
CM-mat-Mono
CF-mat-Mono
d
PE-C3 (82)
CF-C0 (407)
EC-C2 (247)
CPC-C2 (270)
CM-C1 (26)
CSC-C0 (200)
e
Promoter His-RaMA (21047)
Gene Body His-RaMA (19106)
scESC RaMA (160)
f
H3K4me3-Total (12375)
H3K9ac-Total (20728)
H3K27ac-Total (18498)
scESC-RaMA (160)
H3K79me2-Total (8385)
g
H3K79me3 (13380)
H3K79me2 (8385)
H3K36me3 (17008)
scESC-RaMA (160)

Supplementary Fig. 4| RaMA genes exhibit lineage specificity and expression is regulated by distinct histone modifications of monoallelic genes. **a**, Schematic of RaMA gene selection criteria. Genes fit into the selection criteria ‘A’ is considered as RaMA genes. **b**, Percentages and the fold changes of the expressed RaMA genes (bulk-RNA-seq). Percentages of the RaMA genes in each cells type were calculated over the total genes expressed and the fold change of the expressed RaMA genes in CPCCs was calculated over the RaMA genes from mESCs. **c**, UpSet diagram illustrationg the intersects of unique and common RaMA genes in cardiac lineage cell types. RaMA genes are preferentially unique to cell types but shared less between cell types. **d**, Intersects between CSC and CPC RaMA genes and monoallelic genes of four cell types in CBs. Nodes show the intersected gene size and the lines connecting the nodes shows the cell types that are sharing the intersected genes. **e-g**, Assesment of RaMA gene’s association with gene enriched for histone marks identified in CUT&Tag assay. 85.6% of RaMA genes (from mESCs scRNA-seq data) are associated with in-silico modeled histone signatures for promoters and gene bodies (**e**). Venn diagrams showing individual promoter histone signature (**f**) and gene body (**g**) association with RaMA genes.

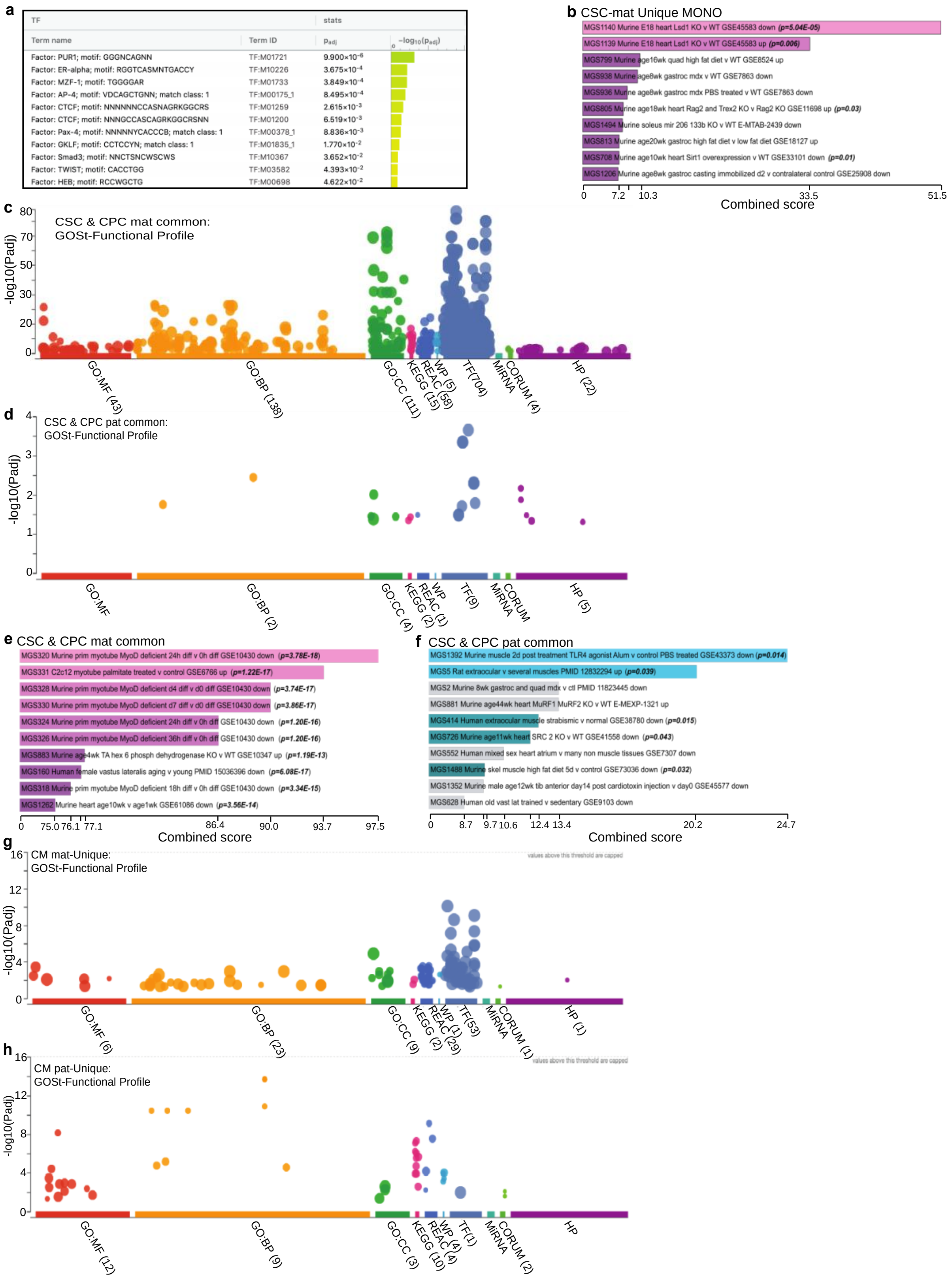
a
TF
stats
Term name
Term ID
Padj
−log10(Padj)
Factor: PUR1; motif: GGGNCAGNN TF:M01721 9.900×10⁻⁶
Factor: ER-alpha; motif: RGGTCASMNTGACCY TF:M10226 3.675×10⁻⁴
Factor: MZF-1; motif: TGGGGAR TF:M01733 3.849×10⁻⁴
Factor: AP-4; motif: VDCAGCTGNN; match class: 1 TF:M00175_1 8.495×10⁻⁴
Factor: CTCF; motif: NNNNNNNCCASNAGRKGGCRS TF:M01259 2.615×10⁻³
Factor: CTCF; motif: NNNGCCASCAGRKGGCRSNN TF:M01200 6.519×10⁻³
Factor: Pax-4; motif: NNNNNYCACCCB; match class: 1 TF:M00378_1 8.836×10⁻³
Factor: GKLF; motif: CCTCCYN; match class: 1 TF:M01835_1 1.770×10⁻²
Factor: Smad3; motif: NNCTSNCWSCWS TF:M10367 3.652×10⁻²
Factor: TWIST; motif: CACCTGG TF:M03582 4.393×10⁻²
Factor: HEB; motif: RCCWGCTG TF:M00698 4.622×10⁻²
b CSC-mat Unique MONO
MGS1140 Murine E18 heart Lsd1 KO v WT GSE45583 down (p=5.04E-05)
MGS1139 Murine E18 heart Lsd1 KO v WT GSE45583 up (p=0.006)
MGS799 Murine age16wk quad high fat diet v WT GSE8524 up
MGS938 Murine age8wk gastroc mdx v WT GSE7863 down
MGS936 Murine age8wk gastroc mdx PBS treated v WT GSE7863 down
MGS805 Murine age18wk heart Rag2 and Trex2 KO v Rag2 KO GSE11698 up (p=0.03)
MGS1494 Murine soleus mir 206 133b KO v WT E-MTAB-2439 down
MGS813 Murine age20wk gastroc high fat diet v low fat diet GSE18127 up
MGS708 Murine age10wk heart Sirt1 overexpression v WT GSE33101 down (p=0.01)
MGS1206 Murine age8wk gastroc casting immobilized d2 v contralateral control GSE25908 down
0 7.2 10.3 33.5 51.5
Combined score
c
CSC & CPC mat common:
GOSt-Functional Profile
-log10(Padj)
80 70 50 30 20 0
GO:MF (43)
GO:BP (138)
GO:CC (111)
KEGG (15)
REAC (58)
WP (5)
TF(704)
MIRNA
CORUM (4)
HP (22)
d
CSC & CPC pat common:
GOSt-Functional Profile
-log10(Padj)
4 3 2 1 0
GO:MF
GO:BP (2)
GO:CC (4)
KEGG (2)
REAC (1)
WP
TF(9)
MIRNA
CORUM
HP (5)
e CSC & CPC mat common
MGS320 Murine prim myotube MyoD deficient 24h diff v 0h diff GSE10430 down (p=3.78E-18)
MGS331 C2c12 myotube palmitate treated v control GSE6766 up (p=1.22E-17)
MGS328 Murine prim myotube MyoD deficient d4 diff v d0 diff GSE10430 down (p=3.74E-17)
MGS330 Murine prim myotube MyoD deficient d7 diff v d0 diff GSE10430 down (p=3.86E-17)
MGS324 Murine prim myotube MyoD deficient 24h diff v 0h diff GSE10430 down (p=1.20E-16)
MGS326 Murine prim myotube MyoD deficient 36h diff v 0h diff GSE10430 down (p=1.20E-16)
MGS883 Murine age4wk TA hex 6 phosph dehydrogenase KO v WT GSE10347 up (p=1.19E-13)
MGS160 Human female vastus lateralis aging v young PMID 15036396 down (p=6.08E-17)
MGS318 Murine prim myotube MyoD deficient 18h diff v 0h diff GSE10430 down (p=3.34E-15)
MGS1262 Murine heart age10wk v age1wk GSE61086 down (p=3.56E-14)
0 75.0 76.1 77.1 86.4 90.0 93.7 97.5
Combined score
f CSC & CPC pat common
MGS1392 Murine muscle 2d post treatment TLR4 agonist Alum v control PBS treated GSE43373 down (p=0.014)
MGS5 Rat extraocular v several muscles PMID 12832294 up (p=0.039)
MGS2 Murine 8wk gastroc and quad mdx v ctl PMID 11823445 down
MGS881 Murine age44wk heart MuRF1 MuRF2 KO v WT E-MEXP-1321 up
MGS414 Human extraocular muscle strabismic v normal GSE38780 down (p=0.015)
MGS726 Murine age11wk heart SRC 2 KO v WT GSE41558 down (p=0.043)
MGS552 Human mixed sex heart atrium v many non muscle tissues GSE7307 down
MGS1488 Murine skel muscle high fat diet 5d v control GSE73036 down (p=0.032)
MGS1352 Murine male age12wk tib anterior day14 post cardiotoxin injection v day0 GSE45577 down
MGS628 Human old vast lat trained v sedentary GSE9103 down
0 8.7 9.7 10.6 12.4 13.4 20.2 24.7
Combined score
g
CM mat-Unique:
GOSt-Functional Profile
values above this threshold are capped
-log10(Padj)
16 12 8 4 0
GO:MF (6)
GO:BP (23)
GO:CC (9)
KEGG (2)
REAC (29)
WP (1)
TF(53)
MIRNA
CORUM (1)
HP (1)
h
CM pat-Unique:
GOSt-Functional Profile
values above this threshold are capped
-log10(Padj)
16 12 8 4 0
GO:MF (12)
GO:BP (9)
GO:CC (3)
KEGG (10)
REAC (4)
WP (4)
TF(1)
MIRNA
CORUM (2)
HP

Supplementary Fig. 5| Critical cardiac genes are DeMA. a, Transcription factor binding motif enrichment of pat-mono genes in CPCs. GOSt profiles shows only TF binding functional profiles for CPC mat-mono genes. **b,** Unique CSC maternal monoallelic genes enrichment in known cardiac muscle development gene sets. **c**,**d**, GOSt functional profiles of CSCs' and CPCs, common mat-mono (**c**) and common pat-mono (**d**) genes. MF, BP, CC and TF shows significantly higher GOSt profiles for mat-mono genes than pat-mono genes. **e**,**f**, Enrichment of CSCs, and CPCs, common mat-mono (**e**) and pat-mono (**f**) genes in SysMyo muscle data. Data indicates higher enrichment of common monoallelic genes in embryonic, neonatal and adult heart. **g**,**h**, Functional profiles of unique maternal and paternal monoallelic genes in CMs. Only one TF (TF:M08998) was enriched for 135 pat-mono genes. Statistical tests used in data analysis: In Fig. 4a, c, d, g, h, - The p-value is computed from the Fisher exact test assuming binomial distribution and independence for probability of any gene belonging to any set. In Fig. 4b, e, f - Combined score was computed by using the log of the P-value from the Fisher exact test and multiplying that by the z-score of the deviation from the expected rank.

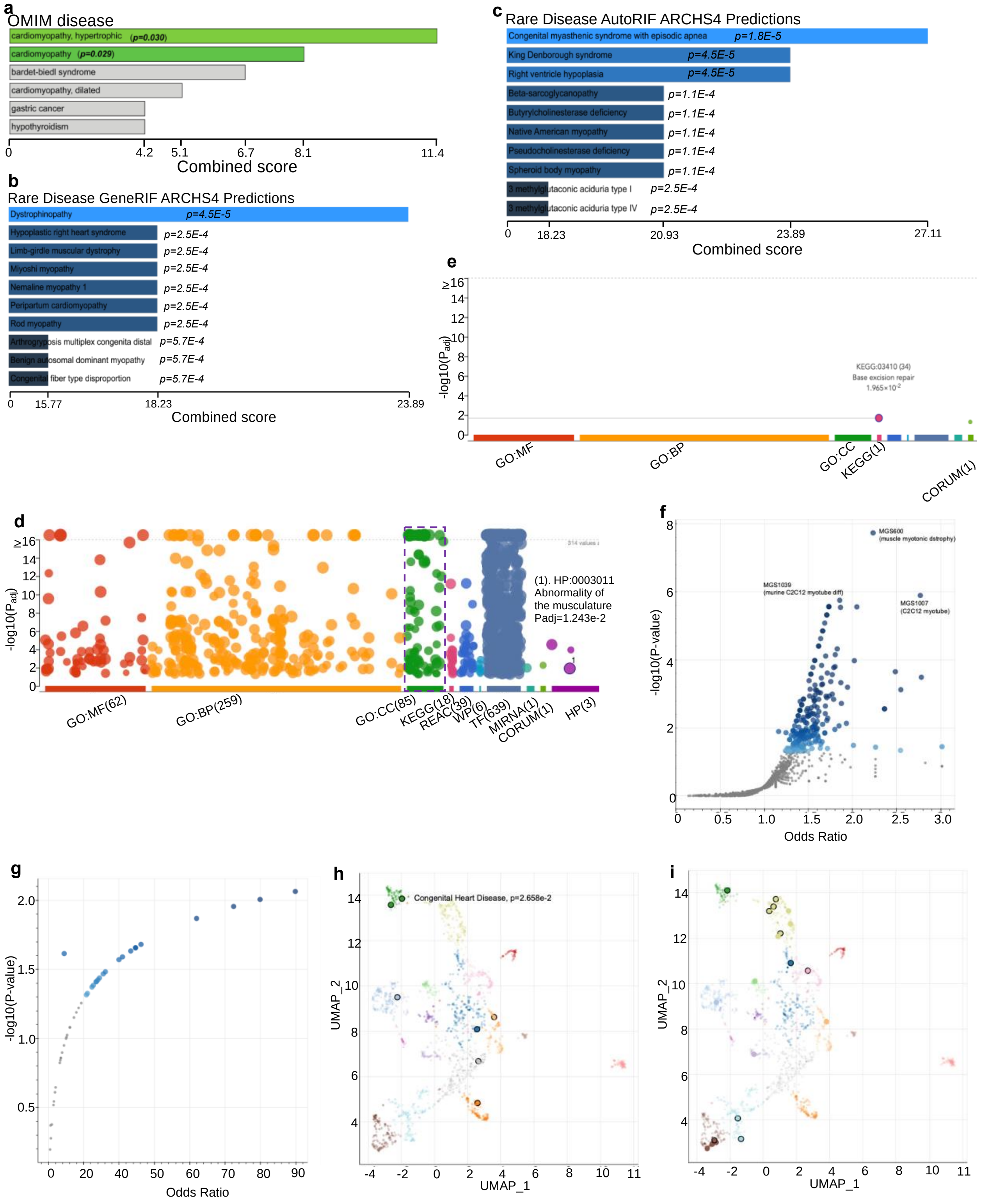

Supplementary Fig. 6| RaMA and DeMA genes disease relevance in heart. **a-c**, CMs' mat-mono genes' involvement in different rare disease categories. Significant involvements were

curated from OMIM disease, Rare disease. Bars represent combined scores. **d**,**e**, Functional profile analysis of DeMA and RaMA genes in CMs. DeMA genes exhibi broader functional profile **(d)** than the RaMA genes **(e)** and DeMA genes are included in 85 cellular component GO terms in which there are processes included for cardiac cell composition build. Among the total DeMA gene set 718 genes (out of 1201 query size) are included in the 'Abnormality of the musculature' human phenotypes. **f**,**g**, Volcano plot showing enrichment of total DeMA gene and RaMA genes in CMs in 'Sysmyo muscle data'. DeMA genes are significantly involved in cardiac cell composition related processes **(f)** while RaMA genes are involving in cellular housekeeping processes **(g)**. **h**,**i** UMAP plots showing DeMA (**h**) and RaMA (**i**)gene's known clinical relevance. The gene sets were compiled with 'Jensen DISEASE', a clinical literature data mining algorithm. DeMA genes are significantly (p=2.658e-2) enriched in clinical congenital heart disease. Statistical tests used in data analysis: In Fig. 6d, e, f, g, h, i - The p-value is computed from the Fisher exact test assuming binomial distribution and independence for probability of any gene belonging to any set. In Fig. 6a, b, c - Combined score was computed by using the log of the P-value from the Fisher exact test and multiplying that by the z-score of the deviation from the expected rank.
