# Supplementary material for: Allele-specific differential regulation of monoallelically expressed autosomal genes in the cardiac lineage
Source: Nat Commun. 2022 Oct 10;13:5984. doi: 10.1038/s41467-022-33722-x (PMC9550772; doi:10.1038/s41467-022-33722-x)
Supplement: Supplementary file 2 — Description of Additional Supplementary Files [file 41467_2022_33722_MOESM2_ESM.pdf]

Description of additional supplementary files

Supplementary Data

File Name: Supplementary Data 1

Description: Histone modification information for deterministic monoallelic genes.

File Name: Supplementary Data 2

Description: Histone modification information for the random autosomal monoallelic genes.

File Name: Supplementary Data 3

Description: GOSt analysis of deterministic monoallelic genes.

File Name: Supplementary Data 4

Description: Deterministic monoallelic gene sets and their correlation to SysMyo muscle data.

File Name: Supplementary Data 5

Description: GOSt analysis of deterministic monoallelic genes in cardiomyocytes.

File Name: Supplementary Data 6

Description: GOSt analysis of random autosomal monoallelic genes in cardiomyocytes.

File Name: Supplementary Data 7

Description: Clinical relevance of deterministic monoallelic genes in cardiomyocytes.

File Name: Supplementary Data 8

Description: Clinical relevance of random autosomal monoallelic genes in cardiomyocytes.

Supplementary Movie

File Name: Supplementary Movie 1

Description: Rhythmically beating cardiac body

Source Data

File Name: Source Data 1

Description: Maternally expressed total DeMA genes in bulk-ESCs

File Name: Source Data 2

Description: Paternally expressed total DeMA genes in bulk-ESCs

File Name: Source Data 3

Description: Maternally actively transcribing DeMA genes in bulk-ESCs

File Name: Source Data 4

Description: Paternally actively transcribing DeMA genes in bulk-ESCs

File Name: Source Data 5

Description: Maternally expressed total DeMA genes in bulk-CPCCs

File Name: Source Data 6

Description: Paternally expressed total DeMA genes in bulk-CPCCs

File Name: Source Data 7

Description: Maternally actively transcribing DeMA genes in bulk-CPCCs

File Name: Source Data 8

Description: Paternally actively transcribing DeMA genes in bulk-CPCCs

File Name: Source Data 9

Description: Maternally expressed total DeMA genes in scESCs

File Name: Source Data 10

Description: Paternally expressed total DeMA genes in scESCs

File Name: Source Data 11

Description: Maternally actively transcribing DeMA genes in scESCs

File Name: Source Data 12

Description: Paternally actively transcribing DeMA genes in scESCs

File Name: Source Data 13

Description: Maternally expressed total DeMA genes in scCSC

File Name: Source Data 14

Description: Paternally expressed total DeMA genes in scCSC

File Name: Source Data 15

Description: Maternally actively transcribing DeMA genes in scCSC

File Name: Source Data 16

Description: Paternally actively transcribing DeMA genes in scCSC

File Name: Source Data 17

Description: Maternally expressed total DeMA genes in scCPC

File Name: Source Data 18

Description: Paternally expressed total DeMA genes in scCPC

File Name: Source Data 19

Description: Maternally actively transcribing DeMA genes in scCPC

File Name: Source Data 20

Description: Paternally actively transcribing DeMA genes in scCPC

File Name: Source Data 21

Description: Maternally expressed total DeMA genes in scCF

File Name: Source Data 22

Description: Paternally expressed total DeMA genes in scCF

File Name: Source Data 23

Description: Maternally actively transcribing DeMA genes in scCF

File Name: Source Data 24

Description: Paternally actively transcribing DeMA genes in scCF

File Name: Source Data 25

Description: Maternally expressed total DeMA genes in scCM

File Name: Source Data 26

Description: Paternally expressed total DeMA genes in scCM

File Name: Source Data 27

Description: Maternally actively transcribing DeMA genes in scCM

File Name: Source Data 28

Description: Paternally actively transcribing DeMA genes in scCM

File Name: Source Data 29

Description: Maternally expressed total DeMA genes in scPE

File Name: Source Data 30

Description: Paternally expressed total DeMA genes in scPE

File Name: Source Data 31

Description: Maternally actively transcribing DeMA genes in scPE

File Name: Source Data 32

Description: Paternally actively transcribing DeMA genes in scPE

File Name: Source Data 33

Description: Maternally expressed total DeMA genes in scEC

File Name: Source Data 34

Description: Paternally expressed total DeMA genes in scEC

File Name: Source Data 35

Description: Maternally actively transcribing DeMA genes in scEC

File Name: Source Data 36

Description: Paternally actively transcribing DeMA genes in scEC

File Name: Source Data 37

Description: RaMA genes expressed in Bulk-ESC

File Name: Source Data 38

Description: RaMA genes expressed in Bulk-CPCCs

File Name: Source Data 39

Description: RaMA genes expressed in scESC

File Name: Source Data 40

Description: RaMA genes expressed in scCSC

File Name: Source Data 41

Description: RaMA genes expressed in scCPC

File Name: Source Data 42

v: RaMA genes expressed in scCF

File Name: Source Data 43

Description: RaMA genes expressed in scCM

File Name: Source Data 44

Description: RaMA genes expressed in scEC

File Name: Source Data 45

Description: RaMA genes expressed in scPE

File Name: Source Data 46

Description: H3K4me1 associated with maternal alleles in ESCs

File Name: Source Data 47

Description: H3K4me1 associated with paternal alleles in ESCs

File Name: Source Data 48

Description: H3K4me3 associated with maternal alleles in ESCs

File Name: Source Data 49

Description: H3K4me3 associated with paternal alleles in ESCs

File Name: Source Data 50

Description: H3K27ac associated with maternal alleles in ESCs

File Name: Source Data 51

Description: H3K27ac associated with paternal alleles in ESCs

File Name: Source Data 52

Description: H3K36me3 associated with maternal alleles in ESCs

File Name: Source Data 53

Description: H3K36me3 associated with paternal alleles in ESCs

File Name: Source Data 54

Description: H3K79me2 associated with maternal alleles in ESCs

File Name: Source Data 55

Description: H3K79me2 associated with paternal alleles in ESCs

File Name: Source Data 56

Description: H3K79me3 associated with maternal alleles in ESCs

File Name: Source Data 57

Description: H3K79me3 associated with paternal alleles in ESCs
