# Supplementary material for: Allele-specific differential regulation of monoallelically expressed autosomal genes in the cardiac lineage
Source: Nat Commun. 2022 Oct 10;13:5984. doi: 10.1038/s41467-022-33722-x (PMC9550772; doi:10.1038/s41467-022-33722-x)
Supplement: Supplementary file 12 — Reporting Summary [file 41467_2022_33722_MOESM12_ESM.pdf]

nature research

Corresponding author(s): Gayan I. Balasooriya and David L. Spector

Last updated by author(s): Sep 12, 2022

Reporting Summary

Nature Research wishes to improve the reproducibility of the work that we publish. This form provides structure for consistency and transparency in reporting. For further information on Nature Research policies, see our Editorial Policies and the Editorial Policy Checklist.

Statistics

For all statistical analyses, confirm that the following items are present in the figure legend, table legend, main text, or Methods section.

n/a	Confirmed	
☐	☒	The exact sample size (*n*) for each experimental group/condition, given as a discrete number and unit of measurement
☐	☒	A statement on whether measurements were taken from distinct samples or whether the same sample was measured repeatedly
☐	☒	The statistical test(s) used AND whether they are one- or two-sided<br>*Only common tests should be described solely by name; describe more complex techniques in the Methods section.*
☒	☐	A description of all covariates tested
☒	☐	A description of any assumptions or corrections, such as tests of normality and adjustment for multiple comparisons
☐	☒	A full description of the statistical parameters including central tendency (e.g. means) or other basic estimates (e.g. regression coefficient) AND variation (e.g. standard deviation) or associated estimates of uncertainty (e.g. confidence intervals)
☐	☒	For null hypothesis testing, the test statistic (e.g. *F*, *t*, *r*) with confidence intervals, effect sizes, degrees of freedom and *P* value noted<br>*Give P values as exact values whenever suitable.*
☒	☐	For Bayesian analysis, information on the choice of priors and Markov chain Monte Carlo settings
☒	☐	For hierarchical and complex designs, identification of the appropriate level for tests and full reporting of outcomes
☒	☐	Estimates of effect sizes (e.g. Cohen's *d*, Pearson's *r*), indicating how they were calculated

Our web collection on statistics for biologists contains articles on many of the points above.

Software and code

Policy information about availability of computer code

Data collection	Single cell RNA sequencing data was collected using Fluidigm® C1™ Single-Cell Auto Prep System (Fluidigm). Agilent 2100 bioanalyzer (Agilent). Illumina HiSeq2500 – V4 flow cell platform (Illumina) for scRNA-Seq. Illumina NextSeq mid output flowcell for Bulk-RNA-seq. qRT-PCR data was collected using QuantStudio3 Real-Time PCR System (ThermoFisher).
Data analysis	Partek® Flow® software (Partek®). ASAP v1 software suite. Seurat 3.1.0 (SeuratV3). Nucleic Acid SeQuence Analysis Resource (NASQAR). StemChecker web-portal. Circa software. Methylomic and epigenomic analysis (MEA) pipeline. SeqMonk bio-informatics web-portal. DeSeq2 (version 1.24.0). InteractiVenn. Intervene shinyApp. GraphPad PRISM (version 7.04). Ensembl BioMart data mining tool. g:Profiler (version e102_eg49_p15_7a9b4d6). Enricher web-portal. Microsoft Office 2011. Adobe Illustrator 2020.

For manuscripts utilizing custom algorithms or software that are central to the research but not yet described in published literature, software must be made available to editors and reviewers. We strongly encourage code deposition in a community repository (e.g. GitHub). See the Nature Research guidelines for submitting code & software for further information.

Data

Policy information about availability of data

All manuscripts must include a data availability statement. This statement should provide the following information, where applicable:

- Accession codes, unique identifiers, or web links for publicly available datasets
- A list of figures that have associated raw data
- A description of any restrictions on data availability

All NGS raw data and processed files generated and used in this study have been uploaded to GEO (GSE173403). Eight Supplementary Data Files, one supplementary Movie file and 57 source data files are submitted with the manuscript. In allele specific gene count tables each cell has two columns, each for

C57BL/6J and CAST/EiJ strains. In the column headings File=cell, number=cell number and B for C57BL/6J and C for CAST/EiJ. For example: File5B means C57BL/6J alleles in 5th cell and File5C means CAST/EiJ alleles in 5th cell. The publicly available data used in this study are; GSM2405897, GSE152103 and GSE80810.

Field-specific reporting

Please select the one below that is the best fit for your research. If you are not sure, read the appropriate sections before making your selection.

☒ Life sciences ☐ Behavioural & social sciences ☐ Ecological, evolutionary & environmental sciences

For a reference copy of the document with all sections, see nature.com/documents/nr-reporting-summary-flat.pdf

Life sciences study design

All studies must disclose on these points even when the disclosure is negative.

Sample size	Three single clones derived from three male F1 mESC lines derived from three blastocysts derived from C57BL6/J female mouse crossed with CAST/EiJ male mouse were used at each experiment point. We did not apply statistical methods to predetermine sample size and followed the general standard practice in the field.
Data exclusions	We did not exclude data.
Replication	We used three biological replicates at each experiment point (one technical replicates per each clone for three clone derived from three blastocysts). All the replicates were successful.
Randomization	We did not include live animals, human subjects or conditions (genetic modifications or screening approaches) in this study therefore randomization was not relevant in this study.
Blinding	We did not include live animals, human subjects or conditions (genetic modifications or screening approaches) in this study therefore blinding was not relevant in this study.

Reporting for specific materials, systems and methods

We require information from authors about some types of materials, experimental systems and methods used in many studies. Here, indicate whether each material, system or method listed is relevant to your study. If you are not sure if a list item applies to your research, read the appropriate section before selecting a response.

Materials & experimental systems

n/a	Involved in the study	
☐	☒	Antibodies
☐	☒	Eukaryotic cell lines
☒	☐	Palaeontology and archaeology
☐	☒	Animals and other organisms
☒	☐	Human research participants
☒	☐	Clinical data
☒	☐	Dual use research of concern

Methods

n/a	Involved in the study	
☐	☒	ChIP-seq
☒	☐	Flow cytometry
☒	☐	MRI-based neuroimaging

Antibodies

Antibodies used	H3K36me3 (Active Motif - Cat. No.61102), H3K4me1 (Active Motif - Cat. No.39298), H2K27ac (Active Motif - Cat. No.39134), H3K4me3 (Active Motif - Cat. No.39160), H3K79me2 (Active Motif - Cat. No.39144), H3K79me3 (Novus Biologicals - Cat. No.NB21-1383SS). We used 1 microgram in 50 microliter dilution for primary antibodies. 1:100 dilution was used for secondary antibogy.
Validation	All antibodies are commercially available, and have been validated either by Western blotting, ChIP assay and previously published CUT&Tag assays. Active Motif antibodies have been validated for CUT&Tag using Active Motif's CUT&Tag-IT™ Assay Kit, Catalog No. 53160. H3K79me3 antibody was raised against epitop surrounding Arginine 8 human histone H3.2 same as in mouse. Tested for ChIP assay in human cells.

Eukaryotic cell lines

Policy information about cell lines

Cell line source(s)	Male F1 mESC lines were generated at the Gene Targeting Core Facility at the Cold Spring Harbor Laboratory.

Authentication	Male F1 mESC lines were authenticated by immunefluorescent staining for Pouf51, Nanog and Sox2 and further validated by bulk and scRNA-seq data.
Mycoplasma contamination	Cells are regularly tested for Mycoplasma contamination. Cell cultures used at all the time points were tested prior to the cell collection. Cells were negative for Mycoplasma.
Commonly misidentified lines (See ICLAC register)	No commonly misidentified cell lines were used in this study.

Animals and other organisms

Policy information about studies involving animals; ARRIVE guidelines recommended for reporting animal research

Laboratory animals	The study did not involve laboratory animals.
Wild animals	n/a
Field-collected samples	n/a
Ethics oversight	No ethical approval or guidance was required. We obtained the mESC lines from Cold Spring Harbor Laboratory (CSHL) Gene Targeting Shared Resource.

Note that full information on the approval of the study protocol must also be provided in the manuscript.

ChIP-seq

Data deposition

☒ Confirm that both raw and final processed data have been deposited in a public database such as GEO.

☒ Confirm that you have deposited or provided access to graph files (e.g. BED files) for the called peaks.

Data access links *May remain private before publication.*	CUT&Tag assay: https://www.ncbi.nlm.nih.gov/geo/query/acc.cgi?acc=GSE173403
Files in database submission	Embryonic Stem Cells - H3K36me3<br>Embryonic Stem Cells - H3K4me1<br>Embryonic Stem Cells - H3K79me2<br>Embryonic Stem Cells - H3K79me3<br>Embryonic Stem Cells - H3K27ac<br>Embryonic Stem Cells - H3K4me3<br>IgG control
Genome browser session (e.g. UCSC)	n/a

Methodology

Replicates	Samples from three biological replicates were pooled to generate each data set.
Sequencing depth	All Experiments were paired-end (300bp). Sequencing depths and sampling is reported in the manuscript.
Antibodies	All antibodies used are provided with their catalog numbers and vendors in the Methods section.
Peak calling parameters	We used SEACR pipeline for CUT&Tag peak calling embedded in Basepair (Version 3.2.7) web portal. Default peak calling parameters were used with no changes.
Data quality	For each data set, data quality assessment was performed using CUT&Tag QC pipeline embedded in Basepair (Version 3.2.7) web portal.
Software	CUT&Tag data was analyzed using Basepair (Version 3.2.7) web portal provided by Active Motif®.
